# Odors and Sensations of Humidity and Dryness in Relation to Sick Building Syndrome and Home Environment in Chongqing, China

**DOI:** 10.1371/journal.pone.0072385

**Published:** 2013-08-26

**Authors:** Juan Wang, Baizhan Li, Qin Yang, Wei Yu, Han Wang, Dan Norback, Jan Sundell

**Affiliations:** 1 Key Laboratory of Three Gorges Reservoir Region’s Eco-Environment, Ministry of Education, Chongqing University, Chongqing, China; 2 Department of Medical Science, Uppsala University and University Hospital, Uppsala, Sweden; 3 Department of Building Science, Tsinghua University, Beijing, China; Duke University, United States of America

## Abstract

The prevalence of perceptions of odors and sensations of air humidity and sick building syndrome symptoms in domestic environments were studied using responses to a questionnaire on the home environment. Parents of 4530 1–8 year old children from randomly selected kindergartens in Chongqing, China participated. Stuffy odor, unpleasant odor, pungent odor, mold odor, tobacco smoke odor, humid air and dry air in the last three month (weekly or sometimes) was reported by 31.4%, 26.5%, 16.1%, 10.6%, 33.0%, 32.1% and 37.2% of the parents, respectively. The prevalence of parents’ SBS symptoms (weekly or sometimes) were: 78.7% for general symptoms, 74.3% for mucosal symptoms and 47.5% for skin symptoms. Multi-nominal regression analyses for associations between odors/sensations of air humidity and SBS symptoms showed that the odds ratio for “weekly” SBS symptoms were consistently higher than for “sometimes” SBS symptoms. Living near a main road or highway, redecoration, and new furniture were risk factors for perceptions of odors and sensations of humid air and dry air. Dampness related problems (mold spots, damp stains, water damage and condensation) were all risk factors for perceptions of odors and sensations of humid air and dry air, as was the presence of cockroaches, rats, and mosquitoes/flies, use of mosquito-repellent incense and incense. Protective factors included cleaning the child’s bedroom every day and frequently exposing bedding to sunshine. In conclusion, adults’ perceptions of odors and sensations of humid air and dry air are related to factors of the home environment and SBS symptoms are related to odor perceptions.

## Introduction

Humans spend 90% of their time in indoor environment and home is the indoor environment where we stay most of our time [1].

Sick building syndrome (SBS) symptoms usually reported by occupants in certain buildings or specific rooms, has been defined by WHO in 1983 [2]. SBS symptoms can be grouped into general symptoms (headache, fatigue, feeling heavy-headed and difficulty concentrating), mucosal symptoms (eye, throat and nose irritations or coughing) and skin symptoms (for example on the face, hands or scalp). Previous studies have shown that SBS is related to personal factors, such as female gender [3], [4], allergies [5] and environmental risk factors, such as building dampness [6], [7], a low ventilation rate [8], indoor air pollution [9], [10], psychosocial factors [11]–[13] and a sensation of dryness [14]. Most SBS studies have focused on office buildings, few are about the home environment [15], [16]. SBS is suggested to be due to sensory irritation appearing in a large fraction of the occupants of the affected building [17].

Perceptions of the indoor air quality include odor perceptions and sensory irritation. An odor (commonly referred to as a smell) is caused by one or more volatile organic compounds (VOCs), usually at very low concentrations, that humans or other animals perceived by the olfactory sense as either pleasant or unpleasant. Odor adaptation is always occurring and can explain individual differences on perceived intensity or quality of special odors [18]. Irritation reflects stimulation of mucosal tissue, chemically-stimulated skin sensation [19], or the trigeminal nerve endings [20]. Perceived irritation on eye and airways may be due to airborne compounds stimulating the sensory nerve endings of the trigeminal nerves [21]. Eye or airway irritation and odor perceptions can be experienced simultaneously and interact with each other. Odor threshold is mostly lower than irritation threshold. Anosmic and normosmic persons have comparable sensitivity to nasal and ocular irritation [22], however, normosmics can detect compounds by olfaction at a much lower level [23], [24]. Complaints of odors may increase subjects’ awareness of underlying symptoms, which may increase symptoms reporting [25].

Most indoor chemicals are nonreactive substances [26]. Formaldehyde is one well studied chemical that cause irritation [27]. Chemical reactive substances are often stronger irritants than non-reactive compounds [21]. Generally, to separate the effects as odorants/irritants of different organic compounds is difficult. Combined effects of sensory irritants in humans can be additive as a first approximation [28].

It is commonly believed that indoor air humidity is of importance for health but the association between humidity perceptions and relative humidity (RH) is not clear. Studies have concluded that increased air pollution levels is a more important factor for the sensation of “dryness” than low relative air humidity [14], [29]. Low indoor air humidity as a risk factor for SBS symptoms and other indoor-related disorders is not clear. Chamber experiments using clean air showed no drying out of the mucosa after exposure to 9% relative humidity for 78 hours [30]. However, Green [31] and Arundel [32] found that dry indoor air could impair the ciliary function, resulting in an increased occurrence of respiratory infections. RH about 40% has been suggested to be better for the eyes and the airways than levels below 30% [33].

Sources of odors in daily life in indoor environments include: human body odors; environmental tobacco smoke (ETS); perfume and cosmetics products; commercial products such as deodorizers and air fresheners and building materials. Moreover, fungal growth can release bio-odorants [34]. A study conducted in Denmark found that 20% of the perceived air pollution in 15 randomly selected offices was due to building materials, 42% to the ventilation system, 25% to indoor smoking and other occupant activities, and 13% to body odor [35].

Ventilation flow in building plays an important role in reducing human exposure to indoor pollutants [36]. Reduced outdoor air flow rate has been shown to be associated with an increase of stuffy odor and poor indoor air quality [8], and a sensation of dry air [37]. A low ventilation rate and thus increased levels of indoor pollutants may also cause SBS symptoms [8]. Source control is needed if the source of odor is strong, especially in combination with less sufficient ventilation.

Odors and air quality perception are early predictors of SBS, and can be a signal for other health effects. It was found that mold odor at the skirting board level was strongly associated with allergic symptoms among children in Sweden [38]. Reporting of odors was also found to be related to asthma symptoms and current cough among adults in another Swedish study [39].

Reports of odors, as well as stuffy air or dry air are common both in homes and other environments [39]–[41]. Engvall found that the most common reported odors were stuffy odor (25.9%) and musty odor (15.6%) in old multifamily dwellings [39].

We have found no previous study from China on odor or humidity perception in relation to the home environment factors or SBS symptoms. The aims of this study include four parts. Firstly to describe the prevalence of perceptions of different odors and air humidity in adults in Chongqing and to study gender differences in these perceptions. Secondly to study associations between these perceptions and SBS in adults in Chongqing. Thirdly to study associations between these perceptions and characteristics of the home environment, in order to identify possible sources of odors or irritative emissions. Lastly to study if associations between home environmental factors and SBS symptoms is different in those perceiving or not perceiving odors or humidity sensations, in order to better understand the role of sensory perception for the effect of different home environment factors for SBS symptoms.

## Materials and Methods

### Ethics Statement

Both the study and the consent procedure were approved by the Medical Research Ethics Committee of School of Public Health, Fudan University. The participants gave informed consent.

### Selection of the Study Subjects

The present study is part of an epidemiological multi-center study of asthma and allergies among children and their relation to the home environment in China (China, Children, Homes, Health, CCHH). The study is using the same study protocol and questionnaire as earlier studies [42], [43], starting with a cross-sectional questionnaire survey followed by a nested case-control study. The survey was carried out during December 2010 to April 2011.

The questionnaires were distributed to children’s parents through teachers in kindergartens in three districts (Shapingba, Jiulongpo, Yubei) that were randomly selected from 9 districts of Chongqing city. From the 54 randomly selected kindergartens (15 from Shapingba, 21 from Jiulongpo and 18 from Yubei), 7117 subjects (one parent per child aged from 1–8 years old) were selected and invited for the questionnaire survey. Completed questionnaires were collected one week later by teachers.

### Questionnaire

A modified version of a self-administered questionnaire previously used in Sweden, and among Chinese university students [42], [43] has been used in this study. The questionnaire was slightly modified to be more appropriate for Chinese culture, lifestyle, building structure and interior characteristics.

Questions about odor and air humidity perceptions were: During the last 3 months, have you had any of the following perceptions: (1) Stuffy odor; (2) Unpleasant odor; (3) Pungent odor; (4) Mold odor; (5) Tobacco smoke odor; (6) Humid air; (7) Dry air. There were 3 options: (A) weekly; (B) sometimes; (C) never.

Questions about SBS symptoms were obtained from the Northern Swedish Office Illness study [37]. They were as follows: During the last 3 months, have you had any of the following symptoms: (1) Fatigue; (2) Feeling heavy headed; (3) Headache; (4) Nausea/Dizziness; (5) Difficulties concentrating; (6) Itching, burning or irritation of the eyes; (7) Irritating, stuffy or runny nose; (8) Hoarse, dry throat; (9) Cough; (10) Dry or flushed facial skin; (11) Scaling/itching scalp or ears; (12) Hands dry, itching, red skin. There were 3 options to choose for each answer: (A) weekly; (B) sometimes; (C) never.

SBS symptoms were grouped into the following three groups: general symptoms, which include fatigue, feeling heavy headed, headache/nausea/dizziness and difficulties concentrating; mucosal symptoms, which include itching, burning or irritation of the eyes, irritating, stuffy or runny nose, hoarse, dry throat and cough; skin symptoms, which include dry or flushed facial skin, scaling/itching scalp or ears and hands dry, itching, red skin. Results for SBS symptoms were analyzed for general symptoms (at least one), mucosal symptoms (at least one), skin symptoms (at least one).

Questions about demographic information, exposure indicators and building characteristics used into the present study included:

(1) Gender;

(2) A history of asthma, allergic rhinitis or eczema (yes/no);

(3) Current smoker (yes/no);

(4) House site (urban/suburban/rural);

(5) Whether current residence is near a main road or highway within a distance of 200 m (yes/no);

(6) Building construction time (before 1980/1980–1990/1991–2000/2001–2005/after 2005);

(7) Residence area (≤40 m^2^/41–60 m^2^/61–75 m^2^/76–100 m^2^/101–150 m^2^/>150 m^2^);

(8) Wall materials on children’s bedroom (wall paper/cement/lime/paint/emulsion paint/other);

(9) Floor materials (wood/cement/ceramic tile or stone/laminated floor/other);

(10) Whether any redecoration has been done since one year before pregnancy (yes/no);

(11) Whether any new furniture has been bought since 1 year before pregnancy (yes/no);

(12) Whether subject has reported any mold spots in child’s bedroom (yes/no);

(13) Whether subject has reported any damp stains in child’s bedroom (yes/no);

(14) Whether subject has reported any water damage in child’s bedroom (yes/no);

(15) Whether subject has reported condensation on window panels during winter in child bedroom (yes/no);

(16) Whether subject has seen cockroaches in home before (yes/no);

(17) Whether subject has seen rats in home before (yes/no);

(18) Whether subject has seen mosquitoes/flies in home before (yes/no);

(19) Whether subject has used mosquito-repellent incense in home before (yes/no);

(20) Whether subject has used incense (not including mosquito-repellent incense) in home before (yes/no);

(21) Whether subject has pets in home currently (yes/no); if yes, please indicate it is (cat/dog/rodent (rabbit/rat)/bird/aquarium fishes or reptiles/other);

(22) The frequency of cleaning child’s bedroom (every day/less than or equal to twice a week);

(23) The frequency of putting child’s bedding to sunshine (frequently/never or rarely);

(24) The frequency of opening window in child’s bedroom in winter (frequently/never or rarely).

### Odor and Humidity Perceptions Score

An odor and humidity perceptions score was constructed (Continuous OH-score, range from 0–7), by adding the number of yes response (weekly or sometimes) to the following odor and humidity perceptions: (1) Stuffy odor; (2) Unpleasant odor; (3) Pungent odor; (4) Mold odor; (5) Tobacco smoke odor; (6) Humid air; (7) Dry air.

The Continuous OH-score was then classified in three categories to make another Categorized OH-score: score category 0 (without any odor or humidity perception), score category 1 (1 out of 7 yes answers), score category 2 (2 or 3 out of 7 yes answers), score category 3 (4 or more out of 7 yes answers).

### Statistical Analysis

All statistical analyses were conducted with SPSS 17.0. Initially, factor analysis was applied to all odor and humidity perceptions questions, using principal component analysis and rotated component matrix (varimax with Kaiser normalisation). Associations between odor and humidity perceptions and SBS symptoms were calculated by multi-nominal regression models with adjustment for parents’ gender, parents’ history of asthma, rhinitis or eczema and current smoker. Then, the same model was applied for stratified analyses (stratified for gender). As a next step, associations between SBS symptoms and the Continuous OH-score were calculated by logistic regression models (enter method). Odds Ratios were calculated for one unit increase on the 7 steps. Stratified analysis was applied (stratified for gender), using the same model. Then, the OH-score was categorized (score0, score1, score2 and score3) and applied in logistic regression models analyzing associations between the Categorized OH-score and SBS symptoms. Stratified analysis was applied (stratified for gender), using the same model. Then, stepwise logistic regression models (forward elimination, condition method) were used to find the most significant variables associated with odors (24 factors from demographic information, exposure indicators and building characteristics). When studying associations between SBS symptoms, home environment and lifestyle characteristics, and odor and humidity perceptions, those reporting any odor (weekly or sometimes) were compared with those not reporting any odor. Results on associations between odor and humidity perceptions and SBS symptoms were given for general symptoms (at least one), mucosal symptoms (at least one), skin symptoms (at least one).

Associations were expressed as odds ratios (OR) with a 95% confidence interval (CI) for logistic regression but relative risk ratios (RRR) with a 95% confidence interval (CI) for multi-nominal regression. Analyses are considered to be statistically significant if the p-value was less than 0.05. In all statistical analysis, two-tailed tests and a 5% level of significance were applied.

## Results

Totally, 5299 of 7117 questionnaires were returned. The total response rate was 74.5%, with small fluctuations across different kindergartens. 4530 complete questionnaires answered by children’ parents (one parent per child) were included in this analysis, excluding questionnaires answered by children’s grandparents or others. Totally, 1340 (29.6%) were males and 3190 (70.4%) were females. Demographic information is shown in Table 1 (percentages for each question are for valid data). Compared with women, men had fewer allergies and were more often smokers. Table 2 shows the prevalence of home environmental characteristics (percentages for each question are for valid data). Table 3 shows the prevalence of odor and humidity perceptions. The most frequently weekly odor was tobacco smoke odor.

**Table 1:** Demographic information on participating parents (n = 4530).

Demographic information	Total (%)	Male (%)	Female (%)	P^a^
A history of asthma, allergic rhinitis or eczema^b^	233(5.5)	45(3.6)	188(6.3)	<0.001
Ever had asthma	69(1.6)	20(1.6)	49(1.6)	0.934
Ever had allergic rhinitis	114(2.7)	20(1.6)	94(3.2)	0.005
Ever had eczema	68(1.6)	11(0.9)	57(1.9)	0.016
Current smoker^c^	708(16.4)	635 (50.8)	73(2.4)	<0.001

a P value by Chi-square test.

b Subjects who have ever had asthma, allergic rhinitis or eczema.

c Subjects who are current smokers.

**Table 2:** Home environmental characteristics of participating parents (n = 4530).

Home environmental characteristics	Subcategory	Result (%)
House site	Urban	3063(71.3)
	Suburban	801(18.6)
	Rural	434(10.1)
Living near a main road or highway^a^	Yes	1846(44.2)
Construction time	Before 1980	154(3.6)
	1980–1990	386(9.1)
	1991–2000	958(22.6)
	2001–2005	1469(34.7)
	After 2005	1268(29.9)
Area	≤40 m^2^	620(14.4)
	41–60 m^2^	573(13.3)
	61–75 m^2^	789(18.4)
	76–100 m^2^	1122(26.1)
	101–150 m^2^	982(22.9)
	>150 m^2^	209(4.9)
Wall material	Wall paper	512(12.0)
	Paint	377(8.8)
	Lime	813(19.0)
	Cement	345(8.1)
	Emulsion paint	2014(47.1)
	other	320(7.5)
Floor material	Wood floor	770(17.7)
	Cement	763(17.5)
	Ceramic tile/stone	1488(34.2)
	Laminated	1225(28.1)
	other	95(2.2)
Redecoration^b^	Yes	1264(34.1)
New furniture^c^	Yes	2334(57.4)
Dampness^d^	Yes	1702(47.3)
Mold spots	Yes	220(5.4)
Damp stains	Yes	342(8.4)
Water damage	Yes	356(9.2)
Condensation	Yes	1273(33.5)
Cockroaches^e^	Yes	3135(76.1)
Rats^e^	Yes	1749(44.3)
Mosquitoes/flies^e^	Yes	3559(85.5)
Current pets	Yes	895(20.8)
Mosquito-repellent incense^f^	Yes	3709(86.7)
Incense^f^	Yes	718(17.1)
Cleaning every day	Yes	1753(41.1)
Frequently put bedding to sunshine	Yes	1753(40.6)
Frequently open window in winter	Yes	1516(35.7)

a Subject’s home located within a distance of 200 meters of a main road or highway.

b Subject’s home has been renovated/redecorated since 1 year before pregnancy.

c Subject’s home has acquired new furniture since 1 year before pregnancy.

d Subject has reported any of the four dampness signs at home: mold spots, damp stains, water damage or condensation on window panels during winter in child’s bedroom.

e Subject has seen cockroaches/rats/mosquitoes/flies or has used mosquito-repellent incense/incense in home.

f Subject has used mosquito-repellent incense/incense in home.

**Table 3:** Odor and humidity perceptions, stratified for gender and a history of asthma, allergic rhinitis or eczema among parents (n = 4530).

			Gender		A history of asthma, allergic rhinitis or eczema	
Category	Frequency	Total (%)	Men (%)	Women (%)	Yes (%)	No (%)
Stuffy odor	Weekly	73(1.8)	12(1.0)	61(2.1)	14(6.6)	52(1.4)
	Sometimes	1264(31.4)	337(29.0)	927(32.4)	81(38.0)	1116(30.7)
Unpleasant odor	Weekly	62(1.6)	14(1.2)	48(1.7)	8(4.0)	48(1.4)
	Sometimes	1023(26.5)	282(25.1)	741(27.0)	66(33.0)	902(25.7)
Pungent odor	Weekly	43(1.1)	12(1.1)	31(1.1)	5(2.4)	34(1.0)
	Sometimes	622(16.1)	163(14.6)	459(16.8)	46(22.4)	533(15.3)
Mold odor	Weekly	31(0.8)	9(0.8)	22(0.8)	3(1.5)	28(0.8)
	Sometimes	406(10.6)	109(9.8)	297(10.9)	33(16.1)	349(10.0)
Tobacco smoke odor	Weekly	293(7.5)	72(6.4)	221(8.0)	33(15.9)	249(7.1)
	Sometimes	1288(33.0)	363(32.1)	925(33.4)	78(37.5)	1138(32.3)
Humid air	Weekly	74(1.9)	19(1.7)	55(2.0)	7(3.4)	61(1.7)
	Sometimes	1244(32.1)	341(30.4)	903(32.8)	78(38.0)	1099(31.4)
Dry air	Weekly	67(1.7)	16(1.4)	51(1.9)	4(2.0)	59(1.7)
	Sometimes	1434 (37.2)	379(34.1)	1055(38.4)	94(46.8)	1278(36.6)

Result from factor analysis of odor and humidity perceptions showed that there were 7 factors, none of the perceptions were grouped together (data not shown).

The prevalence of parents’ SBS symptoms are shown in Table 4. There was no gender difference on the prevalence of SBS symptoms (data not shown). Relative risk ratios for SBS symptoms in relation to odor and humidity perceptions were calculated in multi-nominal regression models with adjustment for gender, a history of asthma, rhinitis or eczema symptoms and current smoker, as shown in Table 5. Stratifying for gender, using the same model as Table 5, showed similar associations between odor and humidity perceptions and SBS symptoms in men and women (data not shown).

**Table 4:** The prevalence of SBS symptoms among participating parents (n = 4530).

SBS symptoms	Weekly orsometimes (%)	Sometimes (%)	Weekly (%)
General symptoms	78.7	65.2	13.5
Mucosal symptoms	74.3	65.8	8.5
Skin symptoms	47.4	42.2	5.2

**Table 5:** Associations between odor and humidity perceptions and SBS symptoms analyzed by multi-nominal regression analyses (n = 4530).

		Odor and humidity perceptions RRR (95% CI)^a^						
SBS symptoms	Frequency	Stuffy odor	Unpleasant odor	Pungent odor	Mold odor	Tobacco smoke odor	Humid air	Dry air
General symptoms	Weekly	3.89(2.96,5.11)***	4.50(3.34,6.05)***	3.09(2.19,4.36)***	2.93(1.97,4.36)***	2.55(1.96,3.32)***	3.14(2.40,4.10)***	2.95(2.27,3.83)***
	Sometimes	2.56(2.06,3.17)***	2.81(2.21,3.57)***	2.12(1.60,2.80)***	1.84(1.33,2.57)***	2.23(1.84,2.71)***	2.03(1.65,2.48)***	2.13(1.75,2.58)***
Mucosal symptoms	Weekly	3.77(2.80,5.07)***	3.40(2.47,4.68)***	3.35(2.33,4.81)***	2.78(1.82,4.26)***	3.02(2.25,4.06)***	2.55(1.89,3.43)***	3.25(2.42,4.37)***
	Sometimes	2.49(2.06,3.02)***	2.64(2.14,3.26)***	2.08(1.62,2.68)***	1.87(1.39,2.52)***	2.32(1.95,2.78)***	1.91(1.60,2.30)***	2.29(1.92,2.74)***
Skin symptoms	Weekly	2.69(1.95,3.71)***	3.13(2.25,4.35)***	3.21(2.23,4.61)***	2.12(1.36,3.31)**	1.73(1.25,2.39)***	2.57(1.86,3.56)***	2.85(2.05,3.97)***
	Sometimes	2.14(1.84,2.49)***	2.22(1.89,2.61)***	2.11(1.74,2.57)***	1.88(1.50,2.36)***	1.61(1.39,1.86)***	1.92(1.65,2.23)***	1.94(1.67,2.24)***

a RRR represents relative risk ratios. This model was adjusted for parents’ gender, parents’ history of asthma, allergic rhinitis or eczema and current smoker. For each type of SBS symptoms (general symptoms/mucosal symptoms/skin symptoms), “never” group was reference category for both “weekly” and “sometimes” group.

*** *P*<0.001, **P<0.01.

Associations between SBS symptoms and the Continuous OH-score were calculated by multiple logistic regression models in Table 6. There were significant associations between the Continuous OH-score and all types of SBS symptoms. Stratifying for gender, using the same model as Table 6, showed similar associations between SBS symptoms and Continuous OH-score in men and women (data not shown).

**Table 6:** Association between SBS symptoms (weekly or sometimes) and Continuous OH-score (as a continuous variable, range from 0–7) (n = 4530).

SBS symptoms	OR (95% CI)^a^	P-value
General symptoms	1.47(1.39,1.57)	<0.001
Mucosal symptoms	1.46(1.38,1.55)	<0.001
Skin symptoms	1.34(1.28,1.39)	<0.001

a Adjusted for parents’ gender, parents’ history of asthma, allergic rhinitis or eczema and current smoker by logistic regression analyses. OR expressed for one unit change of the Continuous OH-score.

Continuous OH-score: by adding the number of yes response (weekly or sometimes) to the following odor and humidity perceptions: (1) Stuffy odor; (2) Unpleasant odor; (3) Pungent odor; (4) Mold odor; (5) Tobacco smoke odor; (6) Humid air; (7) Dry air.

Associations between SBS symptoms and the Categorized OH-score were calculated by multiple logistic regression (Table 7). Results show that the numbers of odor or humidity perceptions were positively associated with SBS symptoms. Stratifying for gender, using the same model as Table 7, showed similar associations between SBS symptoms and Categorized OH-score in men and women (data not shown).

**Table 7:** Associations between SBS symptoms (weekly or sometimes) and Categorized OH-score (n = 4530).

SBS symptoms	CategorizedOH-score	OR (95% CI) a	P-value
General symptoms	Score category 0	1.00	–
	Score category 1	1.86(1.48,2.34)	<0.001
	Score category 2	3.62(2.90,4.53)	<0.001
	Score category 3	5.68(4.14,7.78)	<0.001
Mucosal symptoms	Score category 0	1.00	–
	Score category 1	2.18(1.75,2.71)	<0.001
	Score category 2	3.42(2.78,4.19)	<0.001
	Score category 3	6.11(4.58,8.16)	<0.001
Skin symptoms	Score category 0	1.00	–
	Score category 1	1.86(1.50,2.30)	<0.001
	Score category 2	2.78(2.30,3.36)	<0.001
	Score category 3	4.59(3.67,5.73)	<0.001

a Adjusted for gender, whether had asthma, allergic rhinitis or eczema and current smoker by logistic regression analyses.

Categorized OH-score: score category 0 (without any odor or humidity perception), score category 1 (1 out of 7 yes answers), score category 2 (2 or 3 out of 7 yes answers), score category 3 (4 or more out of 7 yes answers).

As a next step, associations between building characteristics and odor and humidity perceptions were analyzed. 64.6% of the homes in our study were constructed after 2000. Crude analysis showed that older buildings (constructed before 2001) had more stuffy odor [OR (95% CI): 1.16(1.002, 1.33)], unpleasant odor [OR (95% CI): 1.20(1.04,1.40)], mold odor [OR (95% CI): 1.50(1.22,1.84)], tobacco smoke odor [OR (95% CI): 1.21(1.06,1.39)] and the sensation of humid air [OR (95% CI): 1.18(1.03,1.36)]. Stepwise logistic regression (forward elimination) was applied to reduce the models, including the 24 factors (see material and methods) in Table 8 and Table 9. Compared with urban areas, those living in suburban and rural areas reported more mold odor. Females reported more stuffy smell, tobacco smoke and the perception of dry air. Current smoker was associated with tobacco smoke odor, the perception of humid air and dry air, and it was more commonly reported by females. Living near main road or highway, new furniture, dampness indicators (mold spots, damp stains, water damage and condensation), the presence of cockroaches, rats, mosquitoes/flies, and using mosquito-repellent incense and incense were common risk factors for different types of perceptions of impaired air quality. Cleaning every day is a protective factor for stuffy odor. Frequently put bedding to sunshine was a protective factor for stuffy odor, unpleasant odor and pungent odor. Frequently open window in winter was a protective factor for stuffy odor.

**Table 8:** Significant variables associated with odor perception (weekly or sometimes), obtained by forward stepwise logistic regression (n = 4530).

Type of odor	Significant variables	OR (95% CI)	P-value
Stuffy odor	Male gender	0.78(0.62,0.98)	0.033
	Area < = 75m^2^	1.25(1.01,1.55)	0.044
	New furniture	1.29(1.04,1.61)	0.021
	Mold spots	1.86(1.00,3.47)	0.050
	Damp stains	1.87(1.11,3.16)	0.018
	Water damage	1.47(1.03,2.12)	0.036
	Condensation	1.52(1.22,1.89)	<0.001
	Cockroaches	1.53(1.18,2.00)	0.002
	Rats	1.38(1.10,1.72)	0.005
	Mosquitoes/Flies	1.54(1.10,2.16)	0.012
	Incense	1.55 (1.18,2.03)	0.002
	Cleaning every day	0.73(0.59,0.91)	0.006
	Frequently put bedding to sunshine	0.76(0.62,0.95)	0.015
	Frequently open window in winter	0.53(0.42,0.66)	<0.001
Unpleasant odor	Living near main road or highway	1.37(1.09,1.71)	0.007
	Wall materials		0.001
	Wall paper	1.00	–
	Cement	1.87(1.08,3.26)	0.027
	Lime	2.28(1.46,3.57)	<0.001
	Paint	1.94(1.17,3.23)	0.010
	Emulsion paint	1.37(0.93,2.01)	0.117
	New furniture	1.38(1.09,1.74)	0.007
	Mold spots	2.03(1.24,3.34)	0.005
	Water damage	1.49(1.03,2.16)	0.036
	Condensation	1.56(1.24,1.96)	0.001
	Cockroaches	2.07(1.53,2.80)	<0.001
	Rats	1.35(1.06,1.71)	0.013
	Mosquitoes/flies	1.65(1.13,2.40)	0.009
	Incense	1.35(1.004,1.80)	0.047
	Frequently put bedding to sunshine	0.64(0.51,0.81)	<0.001
Pungent odor	Living near main road or highway	1.35(1.03,1.76)	0.028
	Condensation	1.51(1.16,1.98)	0.003
	Cockroaches	1.45(1.03,2.06)	0.035
	Rats	1.82(1.39,2.39)	<0.001
	Frequently put bedding to sunshine	0.65(0.49,0.85)	0.002
Mold odor	House site		0.013
	Urban	1.00	–
	Suburban	1.57(1.03,2.38)	0.035
	Rural	1.89(1.14,3.13)	0.013
	Damp stains	4.28(2.71,6.76)	<0.001
	Water damage	1.71(1.06,2.76)	0.028
	Condensation	1.53(1.09,2.14)	0.013
	Cockroaches	1.65(1.04,2.63)	0.034
	Rats	1.87(1.32,2.65)	<0.001
	Mosquito-repellent incense	2.62(1.24,5.50)	0.011
	Incense	2.04(1.38,3.00)	<0.001
Tobacco smoke odor	Male gender	0.42(0.32,0.56)	<0.001
	A history of asthma, rhinitis or eczema	2.00(1.35,2.96)	0.001
	Current smoker	5.41(3.86,7.60)	<0.001
	New furniture	1.50 (1.23,1.84)	<0.001
	Cockroaches	1.38(1.10,1.74)	0.006
	Rats	1.36(1.11,1.68)	0.004
	Mosquito-repellent incense	1.65(1.20,2.26)	0.002

**Table 9:** Significant variables associated with humidity perception (weekly or sometimes), obtained by forward stepwise logistic regression (n = 4530).

Humidity perception	Significant variables	OR (95% CI)	P-value
Humid air	Current smoker	1.34(1.02,1.74)	0.034
	Wall materials		0.010
	Wall paper	1.00	–
	Cement	0.86(0.52,1.44)	0.573
	Lime	1.32(0.89,1.95)	0.165
	Paint	1.27(0.81,1.99)	0.304
	Emulsion paint	0.83(0.60,1.15)	0.267
	Redecoration	1.50 (1.20,1.88)	<0.001
	Damp stains	3.52(2.34,5.30)	<0.001
	Water damage	1.47(1.03,2.12)	0.035
	Condensation	2.44(1.98,3.02)	<0.001
	Cockroaches	1.58(1.22,2.04)	<0.001
	Rats	1.45(1.16,1.82)	0.001
	Incense	1.45(1.10,1.90)	0.008
Dry air	Male gender	0.63(0.48,0.82)	0.001
	Current smoker	1.78(1.30,2.44)	<0.001
	Floor materials		0.019
	Ceramic tile/Stone	1.00	–
	Cement floor	1.32(0.98,1.78)	0.064
	Wood floor	0.84(0.63,1.11)	0.207
	Laminated floor	0.84(0.66,1.07)	0.158
	New furniture	1.30(1.07,1.59)	0.009
	Condensation	1.52(1.25,1.86)	<0.001
	Cockroaches	1.41(1.13,1.77)	0.003
	Mosquitoes/Flies	1.34(1.01,1.78)	0.041

In total, 74.5% subjects reported at least one odor or humidity perception in the last three months (shorted as “odor group” in the following text) and 25.5% did not report any odor or humidity perception (shorted as “non-odor group” in the following text). Stratified analyses (stratified for odor and humidity perceptions) using stepwise logistic regression models (forward method, condition) were applied to find factors that remained in the models in each stratified group (data not shown). Logistic models were then applied for all factors significant in at least one model in either odor group or non-odor group for particular SBS symptoms (Table 10). A comparison of odds ratios between the odor and non-odor groups showed that there were no great differences, but some factors were more strongly associated with SBS symptoms in the odor group (e.g. new furniture, dampness problems and cleaning every day) while other associations were stronger in the non-odor group (e.g. cockroaches, mosquitoes/flies).

**Table 10:** Significant variables associated with SBS symptoms (weekly or sometimes), stratified for odor or humidity perception.

		Odor group (n = 3031) a		Non-odor group (n = 1049)^a^	
SBS symptoms	Variables	OR (95% CI) b	P-value	OR (95% CI)^b^	P-value
General symptoms	A history of asthma, allergic rhinitis or eczema	3.50(1.41,8.70)	0.007	2.76(0.90,8.44)	0.075
	Living near main road or highway	1.43(1.10,1.86)	0.008	1.85(1.32,2.58)	<0.001
	New furniture	1.96(1.51,2.55)	<0.001	1.35(0.98,1.86)	0.066
	Condensation	1.24(0.95,1.63)	0.118	1.84(1.21,2.79)	0.004
	Cockroaches	1.77(1.33,2.35)	<0.001	1.96(1.42,2.72)	<0.001
	Current pets	1.89(1.31,2.72)	0.001	1.09(0.71,1.67)	0.689
	Cleaning every day	0.75(0.58,0.98)	0.032	0.84(0.61,1.15)	0.278
Mucosal symptoms	Living near main road or highway	1.41(1.13,1.76)	0.002	1.48(1.09,1.99)	0.011
	New furniture	1.73(1.39,2.16)	<0.001	1.33(0.99,1.78)	0.058
	Water damage	1.67(1.10,2.55)	0.017	1.94(0.88,4.29)	0.103
	Cockroaches	1.25(0.97,1.62)	0.089	1.56(1.16,2.11)	0.004
	Cleaning every day	0.69(0.55,0.86)	0.001	0.97(0.72,1.30)	0.834
Skin symptoms	Male gender	0.86(0.70,1.06)	0.150	0.64(0.44,0.92)	0.015
	A history of asthma, allergic rhinitis or eczema	2.18(1.45,3.28)	<0.001	2.43(1.05,5.63)	0.039
	Redecoration	1.49(1.22,1.81)	<0.001	1.09(0.76,1.58)	0.633
	Damp stains	1.60(1.15,2.21)	0.005	1.01(0.40,2.55)	0.989
	Cockroaches	1.13(0.90,1.43)	0.282	1.56(1.07,2.26)	0.020
	Rats	1.42(1.17,1.73)	<0.001	0.95(0.65,1.38)	0.785
	Mosquito/flies	1.78(1.35,2.36)	<0.001	2.22(1.36,3.61)	0.001
	Cleaning every day	0.68(0.56,0.82)	<0.001	0.90(0.64,1.26)	0.532

a Missing data on the odor and non-group for 450 subjects.

b Mutual adjustment models including all variables related to the particular symptom group.

## Discussion

Several indoor environmental risk factors were associated with adults’ odor or humidity perceptions. The most significant risk factors were living near a main road or highway, dampness, redecoration, new furniture, the presence of cockroaches, rats, mosquitoes/flies, using mosquito-repellent incense and incense. Protective factors were a higher frequency of cleaning and putting bedding to sunshine. Odors and humidity perceptions were associated with all types of SBS symptoms, both “weekly” and less common symptoms, but associations for weekly symptoms were stronger. A higher number of reported odor or humidity perceptions were associated with a higher odds ratio for SBS symptoms. The associations between new furniture, signs of dampness, cleaning frequency and SBS symptoms were stronger among the odor group, while the association between SBS symptoms and the presence of cockroaches and mosquitoes/flies were stronger among the non-odor group.

Epidemiological studies can be affected by selection bias. In this study, we included all parents from the cross-sectional study, with no prior information on parents’ health status. The sample size of this study was reasonably large, and the response rate was good (74.5%). Thus, selection bias is fairly unlikely. However, since the study population consists of young parents, results may not be representative for the entire adult population in Chongqing.

Recall bias is another potential problem; subjects may overestimate or underestimate their personal symptoms and/or signs of indoor environment risk factors. Recall bias for odors and humidity perceptions, and SBS symptoms should not be a big issue in this study, since the recall period was short (last three months). Information bias, in which subjects are aware that certain factors have previously been identified as risks, is another potential problem. However, the indoor factors studied in this paper (e.g. wall and floor materials, dampness, odors), are not well known as creating indoor health problems among the Chinese population. Moreover, SBS symptoms is not a concept known in China.

The prevalence of weekly perceptions of odors and humidity perceptions was not high in our study, except the perception of weekly tobacco smoke odor, but less frequent perceptions were common (range from 16.1% to 37.2%).

One main finding was that odor and humidity perceptions were associated with SBS symptoms. Associations between odor and humidity perceptions and SBS symptoms has also been demonstrated in previous studies. One Japanese study found an association between mold odor, stuffiness of air and sick house syndrome (defined similar to SBS) [16]. Odors (stuffy bad air, unpleasant odor and passive smoking) and dry air were found to be significantly associated with SBS symptoms in another Japanese study [44]. A third Japanese study found that mold odor was associated with general symptom (OR = 2.05, p = 0.086) among elementary school pupils [45], and associated with all types of SBS symptoms among residents [46]. A study in office workers reported that the sensation of dryness was strongly associated with the prevalence of SBS symptoms [14]. Brauer et al. found that general symptoms were associated with stuffy air and dry air in a cross-sectional analysis; while, mucosal symptoms were associated with dry air both in cross-sectional analyses and prospective analyses [47]. Moreover, psychosocial factors may influence the air quality perceptions. Perceptions of air dryness and dusty air has been shown to be related with work stress among school personnel [15]. Brauer concluded that it was difficult to determine what existed first: the outcome or the exposure, since symptoms may influence the reporting of environment perceptions [48]. Since our study was cross-sectional, we cannot draw conclusions on causality.

Parents with a history of asthma, allergic rhinitis or eczema had more reports of tobacco smoke odor, but not for other odors or humidity perceptions. This indicates that asthmatic or atopic subjects are more sensitive to some but not all types of odors. Patient studies have demonstrated that nasal allergic symptoms could reduce patients’ sensitivity or olfactory function [49], [50]. Annoyance by tobacco smoke was reported to be more common in asthmatics than in rhinitis patients, which could be explained by a direct chemical influence on the hyper-reactive mucosa [51]. In contrast, one office study found no difference in reporting odor (stuffy air, dry air and unpleasant air) between subjects with or without a history of eczema [52].

A noteworthy finding in our study is that people living near a main road or highway report more unpleasant odor and pungent odor. Complaints of odors could be signal of polluted air. In an Austrian study, 39.7% adult respondents were annoyed by odorous traffic fumes although pollutant levels complied with current World Health Organization (WHO) guidelines [53]. Chongqing is a highly polluted city in China. Our study did not measure the outdoor pollutants, but one study on the air pollutants in and out of the highway toll gates in Chongqing show that average concentrations of indoor and outdoor average concentrations of CO, NO_2_ and PM_10_ exceed the air quality standards [54].

The present study shows that dampness (mold spots, damp stains, water damage or condensation on window panels) was significantly related to perceptions of many types of odor and humidity perceptions. This is in agreement with previous studies. Mold, yeasts, wood-rooting fungi, and bacteria can grow in damp buildings [55]. Building dampness could also increase the emission of VOCs due to degradation of wall/floor materials [56]. Mold odor at the skirting board in dwellings, which can be seen as a proxy for hidden moisture or mold problem in the building structure, has been found to be associated with children’ allergic symptoms [38]. Low ventilation rate in combination with mold odor along the skirting board further increased the risk of children’ asthma or allergies [38]. Mold odor at the skirting board was significantly higher in dorms of asthma cases than controls in a Chinese university dorm study [43]. This local mold odor was also strongly associated with SBS symptoms in the same study [57].

New furniture, which often is of “Pleather” (looks like leather, but is of plastics) in the dwelling was associated with stuffy odor, unpleasant odor, tobacco smoke odor and the perception of dry air in our study and redecoration was associated with the perception of humid air. Formaldehyde concentrations have been shown to be higher in the presence of furniture bought new or restored less than one year before [58]. The results suggested the importance of using low emission materials in indoor environment.

Our study also found that using mosquito-repellent incense in the home was associated with mold odor and tobacco smoke odor and using incense in the home was associated with stuffy odor, unpleasant odor and mold odor. It has been demonstrated that burning one mosquito coil releases the same PM_2.5_ mass as burning 75–137 cigarettes [59]. Incense smoke contains particulate matter (PM), gas products and many organic compounds [60]. Using non-smoke generating mosquito controlling methods and avoid incense smoke exposure could substantially reduce complaints and health risks.

Current smoker was associated with tobacco smoke odor in our study. Smoking is the main source of tobacco smoke odor, but the tobacco smoke odor can contaminate the interior and give tobacco odor. New furniture, the presence of cockroaches, rats, mosquitoes/flies and using mosquito-repellent incense and incense were also found to be associated with tobacco smoke odor in this study. It is unclear why new furniture was associated with tobacco smoke odor, possibly there could be an interaction between the VOCs, or SVOCs (semi-volatile organic compounds) emission and the tobacco smoke.

The presence of cockroaches, rats, mosquitoes/flies were associated with reported odor and humidity perceptions in present study. Such factors could be a reflection of poor hygiene. Stratified analysis on the construction time of building showed that subjects living in older buildings (constructed before 2001) reported significantly more cockroaches, rats, and mosquitoes/flies in homes as well as more stuffy odor, unpleasant odor, mold odor, tobacco smoke odor and the sensation of humid air. However, the associations between construction time of building and odors disappeared when controlling for other building factors.

In this study we found some similar risk factors for the perception of humid air and dry air. They were both associated with current smoker, condensation and the presence of cockroaches, which could be indicators of poor indoor hygiene. Sensation of humid as well as dry air can be indicator of presence of air pollutants rather than a measure of air humidity. Humid air can be an indicator of poor ventilation. It has been discussed that air pollution is a more important factor for the sensation of dryness than low relative humidity [14], [29].

Protective factors were also found in this study, including cleaning every day and frequently putting bedding to sunshine. We found no other study about the association between cleaning frequency and perceptions of odor and humidity in the home environment. However, there is no doubt that cleaning and putting bedding to sunshine could improve indoor hygiene conditions.

Analysis stratified by odor or humidity perception show that there was no great difference between the odor and non-odor group. However, some associations were stronger among the odor group while other factors were more significant in the non-odor group. For the odor group, it seems that new furniture, dampness related factors and cleaning frequency play a more important role of causing SBS symptoms, while, for the non-odor group, cockroaches and mosquitoes/flies could be more important for causing SBS symptoms. SBS symptoms can be caused by different mechanisms, including airway infections, inflammations, allergic reactions or sensory irritations. It has been shown that subjects who were prone to have infections reported more SBS symptoms [61]. Moreover, subjects with elevated levels of biomarkers of allergy and inflammations [62], and sensory irritations [17], [63] had more often SBS symptoms. In our study, factors such as new furniture, signs of dampness and low frequency of cleaning could cause SBS symptoms by sensory irritations since the associations were stronger in the odor group. However, factors such as the presence of cockroaches and mosquitoes/flies could cause SBS symptoms by allergic reactions. More detailed studies are needed to identify biological mechanisms for SBS symptoms.

## Conclusions

Adults’ odor perceptions are associated with both home environmental factors and SBS symptoms. Factors in the home environment, especially living near a main road or highway, dampness and new furniture may increase the risk of odor perception in Chinese residents. The results of this study indicate a need to control material emissions from indoor surfaces, reduce household dampness and encourage frequent cleaning and putting bedding to sunshine to improve occupants’ complaints in indoor environment. Since SBS was associated with odor and humidity perceptions, perceived poor air quality could have medical implications.
